# Supplementary material for: Identification and Expression Profiling of the Regulator of Chromosome Condensation 1 (RCC1) Gene Family in Gossypium Hirsutum L. under Abiotic Stress and Hormone Treatments
Source: Int J Mol Sci. 2019 Apr 8;20(7):1727. doi: 10.3390/ijms20071727 (PMC6479978; doi:10.3390/ijms20071727)
Supplement: Supplementary file 1 [file ijms-20-01727-s001.pdf]

Table S1 Variance analysis of expression level of UVR8 in the wild type (WT) and mutants.

Material name	average value±standard deviation	standard deviation
**WT**	1.0009±0.04883	a
uvr8-1	0.663±0.03577	b
uvr8-2	0.6061±0.0326	b

Table S2 Details of genes co-expressed by Gh_D10G2310 and Gh_A05G3028.

Gene ID	Annotation
Gh_A05G3863	Chloroplastic enzyme responsible for the synthesis of 16:1 fatty acids from galactolipids and sulpholipids. Uses ferredoxin as electron donor.
Gh_A10G2136	Encodes a temperature sensitive plastidic fatty acid desaturase.
Gh_D05G1179	Chloroplastic enzyme responsible for the synthesis of 16:1 fatty acids from galactolipids and sulpholipids. Uses ferredoxin as electron donor.
Gh_D10G2457	Encodes a temperature sensitive plastidic fatty acid desaturase.
Gh_A08G2526	Similar to ACD1. Leaves of antisense ACD1-like plants turn yellow in darkness like wild-type whereas antisense ACD1 plants remain dark after five days of dark treatment.
Gh_D08G2762	Similar to ACD1. Leaves of antisense ACD1-like plants turn yellow in darkness like wild-type whereas antisense ACD1 plants remain dark after five days of dark treatment.
Gh_D08G2764	Similar to ACD1. Leaves of antisense ACD1-like plants turn yellow in darkness like wild-type whereas antisense ACD1 plants remain dark after five days of dark treatment.
Gh_A02G1698	Encodes a peroxisomal catalase, highly expressed in bolts and leaves. mRNA expression patterns show circadian regulation with mRNA levels being high in the subjective early morning. Loss of function mutations have increased H2O2 levels and increased H2O2 sensitivity. Mutants accumulate more toxic ions yet show decreased sensitivity to Li+. This decreased sensitivity is most likely due to an insensitivity to ethylene. Note that in Queval et al. (2007) Plant Journal, 52(4):640, SALK_057998 is named as cat2-1, SALK_076998

	is named as cat2-2; in Bueso et al. (2007) Plant Journal, 52(6):1052, SALK_076998 is named as cat2-1. TAIR has adopted the nomenclature consistent with that in Bueso et al. (2007) after consultation with the authors: SALK_076998 (cat2-1), SALK_057998 (cat2-2).
Gh_A13G1883	Encodes a peroxisomal photorespiratory enzyme that catalyzes transamination reactions with multiple substrates. It is involved in photorespiration.
Gh_D03G0021	Encodes a peroxisomal catalase, highly expressed in bolts and leaves. mRNA expression patterns show circadian regulation with mRNA levels being high in the subjective early morning. Loss of function mutations have increased H2O2 levels and increased H2O2 sensitivity. Mutants accumulate more toxic ions yet show decreased sensitivity to Li+. This decreased sensitivity is most likely due to an insensitivity to ethylene. Note that in Queval et al. (2007) Plant Journal, 52(4):640, SALK_057998 is named as cat2-1, SALK_076998 is named as cat2-2; in Bueso et al. (2007) Plant Journal, 52(6):1052, SALK_076998 is named as cat2-1. TAIR has adopted the nomenclature consistent with that in Bueso et al. (2007) after consultation with the authors: SALK_076998 (cat2-1), SALK_057998 (cat2-2).
Gh_D06G0402	Encodes major plastidic long chain acyl-CoA synthetase with a slight substrate preference of oleic acid over any of the other fatty acids.
Gh_A13G1619	Chloroplastic enzyme responsible for the synthesis of 16:2 and 18:2 fatty acids from galactolipids, sulpholipids and phosphatidylglycerol. Uses ferredoxin as electron donor. Gene mutation resulted in reduced level of unsaturated fatty acids leading to susceptibility to photoinhibition.
Gh_D13G1979	Chloroplastic enzyme responsible for the synthesis of 16:2 and 18:2 fatty acids from galactolipids, sulpholipids and phosphatidylglycerol. Uses ferredoxin as electron donor. Gene mutation resulted in reduced level of unsaturated fatty acids leading to susceptibility to photoinhibition.

Gh_A05G2107	Encodes a protein with putative sucrose-phosphate synthase activity.
Gh_D05G2362	Encodes a protein with putative sucrose-phosphate synthase activity.
Gh_D10G1386	Encodes a protein with putative sucrose-phosphate synthase activity.

Table 3. Expression profiles of genes co-expressed by *Gh_D10G2310* and *Gh_A05G3028* under salt stress.

**Gene**	**leaf_1h_salt_1**	**leaf_1h_salt_2**	**leaf_1h_ck_1**	**leaf_1h_ck_2**	**leaf_3h_salt_1**	**leaf_3h_salt_2**	**leaf_3h_ck_1**	**leaf_3h_ck_2**	**leaf_6h_salt_1**	**leaf_6h_salt_2**	**leaf_6h_ck_1**	**leaf_6h_ck_2**	**leaf_12h_salt_1**	**leaf_12h_salt_2**	**leaf_12h_ck_1**	**leaf_12h_ck_2**
Gh_A05G3863	99.3191	97.8727	1353.18	1453.66	35.6287	35.4819	915.463	955.235	6.61694	6.37459	427.54	439.121	1.3866	1.30225	261.839	278.732
Gh_A10G2136	226.341	225.87	1332.85	1432.83	135.608	136.654	983.062	1033.08	41.148	43.7538	364.5	374.344	16.7826	16.7336	386.707	409.47
Gh_D05G1179	23.1826	22.9742	309.96	320.662	10.8248	10.2262	233.096	217.641	2.46901	2.7011	120.667	106.734	0.924397	0.976686	89.2239	95.3287
Gh_D08G2762	5.18439	4.72141	89.2843	93.2903	210.133	214.992	167.487	170.886	92.5053	91.0629	154.289	152.292	11.8498	12.6519	39.4635	40.6376

Gh_A08G2526	119.072	116.458	192.581	199.791	71.6598	70.721	118.873	119.489	21.4846	23.0648	114.104	111.312	0.861392	0.882537	50.7265	53.021
Gh_D08G2764	90.7098	92.1581	130.452	136.102	2.05141	1.74666	65.2919	66.3736	0.17128	0.0936901	21.2775	21.1932	0.150298	0.112924	3.2471	3.80433
Gh_D10G2457	267.579	274.47	258.832	282.344	64.3723	63.5282	74.3378	74.5283	20.4137	19.9895	46.7217	47.4421	1.91855	1.67682	24.4986	23.5675
Gh_A02G1698	2098.59	2132.87	97.6847	104.047	1081.5	1101.59	49.9447	49.5784	381.588	386.478	24.7055	24.6498	150.443	155.829	4.92082	5.90144
Gh_A05G2107	62.0108	63.4711	17.7676	18.8963	247.652	229.41	42.66	42.9129	44.5793	41.7585	16.2779	15.4586	20.3449	20.5675	7.19627	7.80376
Gh_A13G1619	81.3209	79.7923	56.8465	59.669	1167.77	1180.24	60.1847	62.2164	379.703	389.283	28.8755	26.9356	92.6745	95.8738	18.6346	17.6846
Gh_A13G1883	389.255	358.903	69.518	72.8547	32.7851	32.8827	23.0991	23.0575	6.84489	6.66844	6.42283	6.04902	4.9688	5.33633	10.2853	10.413
Gh_D	2127.	2168.	89.49	95.62	50.37	49.87	23.09	23.05	20.61	20.36	6.422	6.049	7.633	8.273	10.28	10.41

03G0021	03	49	55	08	32	37	91	75	12	65	83	02	21	88	53	3
Gh_D05G2362	46.9856	48.5041	25.9258	25.364	70.6088	65.9869	19.0795	17.8788	28.8219	26.7649	13.2804	12.0855	8.18536	7.62299	15.1622	15.3551
Gh_D06G0402	72.1583	74.6401	56.8465	59.669	22.9432	22.4233	7.05509	7.03218	9.93566	9.21632	5.57019	4.97698	5.83118	5.78738	8.94444	10.3543
Gh_D10G1386	23.3411	22.9949	4.12926	4.42611	22.3365	23.5339	12.56	13.7428	6.52054	6.36039	3.43132	3.67907	2.0294	2.28714	3.88486	3.74238
Gh_D13G1979	101.856	98.2411	30.631	31.7461	8.62531	9.22258	0.640672	1.05594	4.2438	4.3676	1.19018	1.1166	3.40212	3.89637	0.112292	0.249631

Table S4 Details of all *GhRCC1* family genes.

**Gene ID**	**Principle Transcript ID**	**Gene Name**	**Assembly**	**Species**	**Description**	**Chromosome**	**Start**	**End**	**Strand**	**Length (bp)**
**Gh_A01**	Gh_A01	HE	NAU	Gossy	Probable	A01	1,827,	1,833,	+	5,4

**G0184**	G0184.1	RC 1		pium hirsut um	E3 ubiquitin -protein ligase HERC1		663	086		24
**Gh_A02 G0670**	Gh_A02 G0670.1	HE RC 1	NAU	Gossy pium hirsut um	Probable E3 ubiquitin -protein ligase HERC1	A02	11,16 3,214	11,16 9,362	+	6,1 49
**Gh_A03 G0868**	Gh_A03 G0868.1	HE RC 2	NAU	Gossy pium hirsut um	Probable E3 ubiquitin -protein ligase HERC2	A03	51,19 0,168	51,19 6,727	-	6,5 60
**Gh_A03 G1146**	Gh_A03 G1146.1	UV R8	NAU	Gossy pium hirsut um	Ultraviol et-B receptor UVR8	A03	82,09 3,431	82,09 6,182	+	2,7 52
**Gh_A04 G0246**	Gh_A04 G0246.1	UV R8	NAU	Gossy pium hirsut um	Ultraviol et-B receptor UVR8	A04	5,216, 030	5,220, 482	-	4,4 53
**Gh_A05 G2059**	Gh_A05 G2059.1	HE RC 1	NAU	Gossy pium hirsut um	Probable E3 ubiquitin -protein ligase HERC1	A05	22,62 1,736	22,62 8,393	-	6,6 58
**Gh_A05 G2188**	Gh_A05 G2188.1	rcc2	NAU	Gossy pium hirsut um	Protein RCC2 homolog	A05	25,17 5,332	25,17 9,515	-	4,1 84
**Gh_A05 G3028**	Gh_A05 G3028.1	UV R8	NAU	Gossy pium hirsut um	Ultraviol et-B receptor UVR8	A05	77,60 3,269	77,60 8,147	-	4,8 79
**Gh_A07 G0164**	Gh_A07 G0164.1	UV R8	NAU	Gossy pium hirsut um	Ultraviol et-B receptor UVR8	A07	2,075, 273	2,077, 370	+	2,0 98
**Gh_A08**	Gh_A08	HE	NAU	Gossy	Probable	A08	831,6	836,3	-	4,7

**G0100**	G0100.1	RC2		pium hirsutum	E3 ubiquitin-protein ligase HERC2		22	52		31
**Gh_A08G0465**	Gh_A08G0465.1	Herc2	NAU	Gossypium hirsutum	E3 ubiquitin-protein ligase HERC2	A08	6,368,925	6,373,210	+	4,286
**Gh_A09G0062**	Gh_A09G0062.1	UVR8	NAU	Gossypium hirsutum	Ultraviolet-B receptor UVR8	A09	1,367,420	1,375,343	-	7,924
**Gh_A09G1023**	Gh_A09G1023.1	UVR8	NAU	Gossypium hirsutum	Ultraviolet-B receptor UVR8	A09	60,287,669	60,296,340	-	8,672
**Gh_A09G1308**	Gh_A09G1308.1	THO3	NAU	Gossypium hirsutum	THO complex subunit 3	A09	65,714,420	65,718,247	-	3,828
**Gh_A09G2009**	Gh_A09G2009.1	HERC1	NAU	Gossypium hirsutum	Probable E3 ubiquitin-protein ligase HERC1	A09	73,287,778	73,292,152	-	4,375
**Gh_A10G0603**	Gh_A10G0603.1	UVR8	NAU	Gossypium hirsutum	Ultraviolet-B receptor UVR8	A10	8,602,138	8,613,441	-	11,304
**Gh_A10G1652**	Gh_A10G1652.1	UVR8	NAU	Gossypium hirsutum	Ultraviolet-B receptor UVR8	A10	88,054,731	88,063,481	-	8,751
**Gh_A10G2003**	Gh_A10G2003.1	UVR8	NAU	Gossypium hirsutum	Ultraviolet-B receptor UVR8	A10	97,807,087	97,812,175	+	5,089
**Gh_A11G0277**	Gh_A11G0277.1	UVR8	NAU	Gossypium hirsutum	Ultraviolet-B receptor UVR8	A11	2,556,265	2,563,705	-	7,441

**Gh_A11G1355**	Gh_A11G1355.1	UVR8	NAU	Gossypium hirsutum	Ultraviolet-B receptor UVR8	A11	17,373,259	17,375,063	-	1,805
**Gh_A11G1473**	Gh_A11G1473.1	UVR8	NAU	Gossypium hirsutum	Ultraviolet-B receptor UVR8	A11	20,355,133	20,359,642	-	4,510
**Gh_A11G1503**	Gh_A11G1503.1	UVR8	NAU	Gossypium hirsutum	Ultraviolet-B receptor UVR8	A11	21,020,508	21,026,648	+	6,141
**Gh_A11G1567**	Gh_A11G1567.1	HERC1	NAU	Gossypium hirsutum	Probable E3 ubiquitin-protein ligase HERC1	A11	22,786,791	22,791,697	+	4,907
**Gh_A11G2084**	Gh_A11G2084.1	ibtk	NAU	Gossypium hirsutum	Inhibitor of Bruton tyrosine kinase	A11	66,979,397	66,985,120	+	5,724
**Gh_A11G3194**	Gh_A11G3194.1	UVR8	NAU	Gossypium hirsutum	Ultraviolet-B receptor UVR8	scaffold2941_A11	9,171	27,707	+	18,537
**Gh_A13G0645**	Gh_A13G0645.1	UVR8	NAU	Gossypium hirsutum	Ultraviolet-B receptor UVR8	A13	18,020,525	18,024,934	-	4,410
**Gh_A13G1460**	Gh_A13G1460.1	ibtk	NAU	Gossypium hirsutum	Inhibitor of Bruton tyrosine kinase	A13	72,447,838	72,448,784	-	947
**Gh_D01G0233**	Gh_D01G0233.1	HERC1	NAU	Gossypium hirsutum	Probable E3 ubiquitin-protein ligase HERC1	D01	1,989,670	1,995,054	+	5,385
**Gh_D02G0718**	Gh_D02G0718.1	HERC	NAU	Gossypium	Probable E3	D02	10,531,951	10,537,338	+	5,388

		1		hirsutum	ubiquitin-protein ligase HERC1					
**Gh_D02G1249**	Gh_D02G1249.1	UVR8	NAU	Gossypium hirsutum	Ultraviolet-B receptor UVR8	D02	40,138,479	40,144,876	-	6,398
**Gh_D02G1578**	Gh_D02G1578.1	UVR8	NAU	Gossypium hirsutum	Ultraviolet-B receptor UVR8	D02	54,401,729	54,406,922	+	5,194
**Gh_D04G0616**	Gh_D04G0616.1	UVR8	NAU	Gossypium hirsutum	Ultraviolet-B receptor UVR8	D04	11,034,497	11,039,334	+	4,838
**Gh_D05G2301**	Gh_D05G2301.1	HERC1	NAU	Gossypium hirsutum	Probable E3 ubiquitin-protein ligase HERC1	D05	22,364,325	22,371,118	-	6,794
**Gh_D05G2447**	Gh_D05G2447.1	rcc2	NAU	Gossypium hirsutum	Protein RCC2 homolog	D05	24,547,132	24,551,323	-	4,192
**Gh_D05G3007**	Gh_D05G3007.1	UVR8	NAU	Gossypium hirsutum	Ultraviolet-B receptor UVR8	D05	37,819,641	37,823,344	+	3,704
**Gh_D05G3147**	Gh_D05G3147.1	UVR8	NAU	Gossypium hirsutum	Ultraviolet-B receptor UVR8	D05	47,189,594	47,192,537	-	2,944
**Gh_D05G3461**	Gh_D05G3461.1	UVR8	NAU	Gossypium hirsutum	Ultraviolet-B receptor UVR8	D05	56,641,055	56,645,506	+	4,452
**Gh_D06G0015**	Gh_D06G0015.1	rcc2	NAU	Gossypium hirsutum	Protein RCC2 homolog	D06	118,712	123,959	-	5,248
**Gh_D07**	Gh_D07	UV	NAU	Gossy	Ultraviol	D07	2,309,	2,311,	+	2,0

**G0221**	G0221.1	R8		pium hirsutum	et-B receptor UVR8		069	151		83
**Gh_D08 G0551**	Gh_D08 G0551.1	HERC2	NAU	Gossypium hirsutum	Probable E3 ubiquitin-protein ligase HERC2	D08	6,370,686	6,374,970	+	4,285
**Gh_D08 G2751**	Gh_D08 G2751.1	HERC1	NAU	Gossypium hirsutum	Probable E3 ubiquitin-protein ligase HERC1	scaffold4315_D08	11,874	18,909	-	7,036
**Gh_D09 G0059**	Gh_D09 G0059.1	UVR8	NAU	Gossypium hirsutum	Ultraviolet-B receptor UVR8	D09	1,537,993	1,545,883	-	7,891
**Gh_D09 G1044**	Gh_D09 G1044.1	UVR8	NAU	Gossypium hirsutum	Ultraviolet-B receptor UVR8	D09	36,460,186	36,468,799	-	8,614
**Gh_D09 G2222**	Gh_D09 G2222.1	HERC1	NAU	Gossypium hirsutum	Probable E3 ubiquitin-protein ligase HERC1	D09	49,423,794	49,428,157	-	4,364
**Gh_D10 G1050**	Gh_D10 G1050.1	UVR8	NAU	Gossypium hirsutum	Ultraviolet-B receptor UVR8	D10	16,006,114	16,018,438	-	12,325
**Gh_D10 G1907**	Gh_D10 G1907.1	UVR8	NAU	Gossypium hirsutum	Ultraviolet-B receptor UVR8	D10	53,240,175	53,248,825	-	8,651
**Gh_D10 G2310**	Gh_D10 G2310.1	UVR8	NAU	Gossypium hirsutum	Ultraviolet-B receptor UVR8	D10	61,345,236	61,350,325	+	5,090
**Gh_D11 G0331**	Gh_D11 G0331.1	UVR8	NAU	Gossypium hirsut	Ultraviolet-B receptor	D11	2,817,397	2,824,509	-	7,113

				um	UVR8					
**Gh_D11G1503**	Gh_D11G1503.1	UVR8	NAU	Gossypium hirsutum	Ultraviolet-B receptor UVR8	D11	14,948,548	14,950,352	-	1,805
**Gh_D11G1630**	Gh_D11G1630.1	UVR8	NAU	Gossypium hirsutum	Ultraviolet-B receptor UVR8	D11	17,215,939	17,220,442	-	4,504
**Gh_D11G1664**	Gh_D11G1664.1	UVR8	NAU	Gossypium hirsutum	Ultraviolet-B receptor UVR8	D11	17,833,298	17,839,422	-	6,125
**Gh_D11G1726**	Gh_D11G1726.1	HERC1	NAU	Gossypium hirsutum	Probable E3 ubiquitin-protein ligase HERC1	D11	18,961,594	18,966,455	-	4,862
**Gh_D11G2263**	Gh_D11G2263.1	UVR8	NAU	Gossypium hirsutum	Ultraviolet-B receptor UVR8	D11	41,126,628	41,140,544	-	13,917
**Gh_D11G2399**	Gh_D11G2399.1	ibtk	NAU	Gossypium hirsutum	Inhibitor of Bruton tyrosine kinase	D11	47,660,109	47,665,938	+	5,830
**Gh_D13G0764**	Gh_D13G0764.1	UVR8	NAU	Gossypium hirsutum	Ultraviolet-B receptor UVR8	D13	12,545,537	12,549,940	-	4,404
**Gh_D13G1736**	Gh_D13G1736.1	IBTK	NAU	Gossypium hirsutum	Inhibitor of Bruton tyrosine kinase	D13	52,057,426	52,063,226	+	5,801

Table S5 A list of primers used in this study.

**Primers noun**	**Primer sequences**
**Gh_A01G0184F**	CTCCTGGAAAACCTCATCGCGT
**Gh_A01G0184R**	AAGCAGCATCGAATCGAGTCCC
**Gh_A02G0670F**	TTTGGGGATGGTACATTCGGCG
**Gh_A02G0670R**	CCACATGCCACAGCAATCGTTC

**Gh_A03G0868F**	GGGCATGGAACTGGAGTCAGTC
**Gh_A03G0868R**	CCAAATGTGAACAACTGGCCGC
**Gh_A03G1146F**	TGGGTGGAAAGCTTGGACATGG
**Gh_A03G1146R**	AACCACCATTGGCTGGAGGTTC
**Gh_A04G0246F**	ATGTTCTTCGTGTCTCCGCTGG
**Gh_A04G0246R**	TTGCACAAACCTGCACAGCAAG
**Gh_A05G2059F**	CTCGGCTTGTAGACAGGCGTTT
**Gh_A05G2059R**	CGCAAACACGGTAAGGTTTCCC
**Gh_A05G2188F**	TTGTTCTGTGGCTCCACGTGTT
**Gh_A05G2188R**	GATATCAACGCCGACGAGAGGG
**Gh_A05G3028F**	GAACTGGTGGTGATCAAGCCGA
**Gh_A05G3028R**	CGAGCTGGCCATACTTGTTCCA
**Gh_A07G0164F**	AAGGCTGGAGGAATGACTTCGC
**Gh_A07G0164R**	GTTGCCACCCCAAGAGTAGCAT
**Gh_A08G0100F**	ACACGCGAAGAAGTTCACCCTT
**Gh_A08G0100R**	GCGAAGGTGATCTCCACCATCCT
**Gh_A08G0465F**	TGGCATTGACAGAGGATGGACA
**Gh_A08G0465R**	TGGTTTCGGTTTCCAACCACCA
**Gh_A09G0062F**	GGTGGAAGCTTTAGCCGACAGT
**Gh_A09G0062R**	CTTGTTCCATCCCCAGCCGTAG
**Gh_A09G1023F**	ACGGTCGATTAGGTCATGGGGA
**Gh_A09G1023R**	AGTGAAGTTGGTGCCACAAGCA
**Gh_A09G1308F**	GCTTGCTTCCGGTTCTGTCGAT
**Gh_A09G1308R**	TGGCCTATGAACTGCTTTCGCA
**Gh_A09G2009F**	ATCAGTTTGCTCTGGCTGTCGG
**Gh_A09G2009R**	TATCGCATACACGGTGCGGTTT
**Gh_A10G0603F**	TATGGTTGCTTGTGGATGGCGG
**Gh_A10G0603R**	TTGACCATACTTGCTCCACCCG
**Gh_A10G1652F**	CTACTTGTGTTGCCGCTCTGGT
**Gh_A10G1652R**	GCTTGCCCTCAACTCGGATAGG
**Gh_A10G2003F**	GGGCGACACAGTGCAGTAATCA
**Gh_A10G2003R**	TCCTGATGCCCAGTAAGGCAGA
**Gh_A11G0277F**	TGGTCTTCTAGGGCACGGAACT
**Gh_A11G0277R**	ACTTGTGCTACTACGGTCCCCA
**Gh_A11G1355F**	TCTAACCGCCCATCCTGTGTCT
**Gh_A11G1355R**	TGTTTCCCCTTCCCATCCCTCA
**Gh_A11G1473F**	GCACAGCTGCTATTGCAGAACC
**Gh_A11G1473R**	TCCACAGGACACTTGGCGGATA
**Gh_A11G1503F**	GCTGCTGGGGGTACTCATTCTG
**Gh_A11G1503R**	AAGTCCCATCCTCCTGGAGAGC
**Gh_A11G1567F**	GACCCAGAAGGCTCGCAAAAGA
**Gh_A11G1567R**	GCTCGATTTTCCGTCGGCATTC
**Gh_A11G2084F**	AGCTGGGCTATACGTCTGTGGA
**Gh_A11G2084R**	TAGCCAAGTTGACCCTCTCGGT

**Gh_A11G3194F**	AGAGAGCCGGTTGAAGTTTCGG
**Gh_A11G3194R**	AGCATTCCACTTTGGTTGGCCT
**Gh_A13G0645F**	CTCTGCCCCCAAAGTGTAGGTG
**Gh_A13G0645R**	GGGTTGGAACCTTGCCATCAGT
**Gh_A13G1460F**	TCTCCTTTCTGGGCCTGTGTTG
**Gh_A13G1460R**	ACCACTTCCCCAGCTAAACACC
**Gh_D01G0233F**	TCGGCTTCTTCAACGGCATCAT
**Gh_D01G0233R**	GGAGGTGCTGCTTCTGTGGTTT
**Gh_D02G0718F**	ACTGCGGGATGGAACTAGGGAT
**Gh_D02G0718R**	CAGTTTGCCCAGAAATCGACGC
**Gh_D02G1249F**	GCTCTAACAACCTCTGGGCGAG
**Gh_D02G1249R**	TCAACCCGAGTTGGAACCTTGC
**Gh_D02G1578F**	AGCTCCACCGATTTCGATCGTC
**Gh_D02G1578R**	CGGAAAATCGCTGAATCCCCCT
**Gh_D04G0616F**	GGACGACACAGTGCGGTAATCA
**Gh_D04G0616R**	GTCCTGCCGCAACAGACACTAT
**Gh_D05G2301F**	CACGTTCAGCTACCCCCATTCC
**Gh_D05G2301R**	CTCAACCTGTCCACGCAACGTA
**Gh_D05G2447F**	GATGAGTGGGTTCCTCGTCGTG
**Gh_D05G2447R**	TATACAATTGCCCACCGCCTGC
**Gh_D05G3007F**	GTTAGTGATGCTGGGCGAGTGT
**Gh_D05G3007R**	GGGGATGTAGGACATCTCATCGCT
**Gh_D05G3147F**	GCAAATGCAGACCACAGTGCAG
**Gh_D05G3147R**	TCAGATGTAGCCCCAAACCCTCT
**Gh_D05G3461F**	GGGTGTCACGATGTTTGTCCCA
**Gh_D05G3461R**	CCTGCAGAGGTTTGGACCATGT
**Gh_D06G0015F**	AGTCATCTCCCGTCCGTTGTCT
**Gh_D06G0015R**	TGGCCAAGCTGACCATACTGTG
**Gh_D07G0221F**	TTTCCCGGTTCGGGTCAAGTTC
**Gh_D07G0221R**	ATTCCTCCAGCCTTGACTGCAC
**Gh_D08G0551F**	CCTGTGGAGGTTCATCTTCGGC
**Gh_D08G0551R**	CGACTGCAACGGATAGCACGTA
**Gh_D08G2751F**	CGGGGAAGCTATTTACGTGGGG
**Gh_D08G2751R**	CAACTGTCAGACTGTGTCCGCA
**Gh_D09G0059F**	GACAGGTCGGGGTTGGTGAAAA
**Gh_D09G0059R**	TGTGCCTCCATCCACACGAAAC
**Gh_D09G1044F**	TTCCGATCTTAGCAGAACGGGC
**Gh_D09G1044R**	CGTACTTCAGCAGGTATGCCCC
**Gh_D09G2222F**	GGGGCATATCATGTTGCCGTCT
**Gh_D09G2222R**	TGTCCTAGTCTTCCGTTTGCACC
**Gh_D10G1050F**	GTACAAATGGACAGCTCGGGCA
**Gh_D10G1050R**	CTATCTGTTGGCCACTCGAGCC
**Gh_D10G1907F**	GAGGTTTTCTCTTGGGGAGCCG
**Gh_D10G1907R**	AAATTGCCTGAGCTACCACCCC

**Gh_D10G2310F**	TCAGCTTGGTCTTGGTGACGTG
**Gh_D10G2310R**	CGCATGCGATGTTTTTCGGACT
**Gh_D11G0331F**	GGGCGATGGGGAAAGCCTAAAT
**Gh_D11G0331R**	CGGCAGTACGCTGTCCTGATAC
**Gh_D11G1503F**	CGCAGAGTTGAAGCACTTGCAG
**Gh_D11G1503R**	TCGGCTGAATAACCCCAGCCTA
**Gh_D11G1630F**	GCTAGGCAGAGGCGTTACTTCC
**Gh_D11G1630R**	CACAAGCTCAGGAGCCTCATCC
**Gh_D11G1664F**	TGTTATTGCTTGGGGCTCAGGTGAAG
**Gh_D11G1664R**	AACAACTTGCCATCATCACAAATAG
**Gh_D11G1726F**	GCAAGCCTTGTCGCGTTTGTAA
**Gh_D11G1726R**	TCTGTGAGACTGAGGCGTCGAT
**Gh_D11G2263F**	GTTGCAGCTGGGTTGTTGCATT
**Gh_D11G2263R**	GCATGGTTGCTTTCTTGGCCTC
**Gh_D11G2399F**	ATTCGAAGGCTTCTAGCGGCTG
**Gh_D11G2399R**	AGATGACCGAAATGCAGAGCCC
**Gh_D13G0764F**	AGGCGATTAAAGGAGGTCACTGC
**Gh_D13G0764R**	TGTTGGAAGTCTGTTAGCCGCC
**Gh_D13G1736F**	GCAGCAGAGTGTCTAGTGGAGC
**Gh_D13G1736R**	GAACATCTGGTGATGCGCTTGC
**uvr8-1-LP**	TTTGAATATCTCGCTCGATCG
**uvr8-1-RP**	GGAAACTCGATTAAAGGCCAG
**uvr8-2-LP**	TTCCAGATCGGATGAAACAAG
**uvr8-2-RP**	CTTACGAGGAGGAGCCGTAAC
**UVR8-RT-U**	CTTCCGTTGGATTCGAAGGT
**UVR8-RT-L**	ACACAGTTTACAACGCCCAT
**LBb1.3**	ATTTTGCCGATTTCGGAAC
**A05G3863-RT-U**	AAACTCTGGCCATCCAGACTCG
**A05G3863-RT-L**	AAGCCGACACTTTCCCATTGCT
**D05G1179-RT-U**	GCAGAGTCCTTTGGGCTCAAGT
**D05G1179-RT-L**	TGACACCCCATTAGCCAAAGCC
**A13G1619-RT-U**	TATAGGCCACGATTGCGCTCAC
**A13G1619-RT-L**	GTGCCTGTCATGCTTAAACCGC
**D06G0402-RT-U**	GGTGCCACCTTTTCTGAGGTCG
**D06G0402-RT-L**	AGGCAACCATCAGCGTGAAACT
**A02G1698-RT-U**	ACTCAAAGGCACCGTCTTGGAC
**A02G1698-RT-L**	TGCACTTCTCTCTTTTGCCGCT
**A13G1883-RT-U**	CGCGAAATCGGTTCGGGTTTTC
**A13G1883-RT-L**	CTTCAAGCCCCATGCTTCCACT
**D03G0021-RT-U**	AAGAGTAGGTGGCGCGAATCAC
**D03G0021-RT-L**	CAACGGGAAGATGTCCTCTGGC
**D06G0402-RT-U**	GGTGCCACCTTTTCTGAGGTCG
**D06G0402-RT-L**	AGGCAACCATCAGCGTGAAACT
**A05G2107-RT-U**	TGAGGCTTGCTAGGACCGAAGA

**A05G2107-RT-L**	CTGCGCGAGTGTAGACAAGGTT
**D05G2362-RT-U**	ACCTTGTCTACACTCACGCAGC
**D05G2362-RT-L**	GCCACCAAGCAGGTCCTCATAG
**D10G1386-RT-U**	GTGGCAGTGGCAGTGAAGTGTA
**D10G1386-RT-L**	AGTATGCAACACAGTGGGCGTT
**Gh_A05G3028-CDS-U**	acgggggacgagctcggtaccATGAACGGAGAAGGAAAAAAAAGC
**Gh_A05G3028-CDS-L**	gcccttgctcaccatgtcgacGCTGGCTGACTCAGCCAGAA
**Gh_D10G2310-CDS-U**	acgggggacgagctcggtaccATGAATGGAGAAGGAAAAGAAAGTA
**Gh_D10G2310-CDS-L**	gcccttgctcaccatgtcgacGGTGGGTGATTCAGCCAGAAG

Table S6 All primers used in the VIGS experiment.

Primers noun	Primer sequences
Gh_A05G3028-CDS-U	acgggggacgagctcggtaccATGAACGGAGAAGGAAAAAAAAGC
Gh_A05G3028-CDS-L	gcccttgctcaccatgtcgacGCTGGCTGACTCAGCCAGAA
Gh_D10G2310-CDS-U	acgggggacgagctcggtaccATGAATGGAGAAGGAAAAGAAAGTA
Gh_D10G2310-CDS-L	gcccttgctcaccatgtcgacGGTGGGTGATTCAGCCAGAAG
A02G1698-RT-U	ATTCTGGTGCTCCAGTTTGG
A02G1698-RT-L	ACGTTCAGGTATGCGTTCTC
A05G2107-RT-U	TCAAGGACGCCAAATTCCAA
A05G2107-RT-L	ACCTTAACCCATGTCCGGTA
A13G1619-RT-U	ATTGGAGAGCCTCTTCCTGA
A13G1619-RT-L	CACATCGTCAATCTCAAACACC
A13G1883-RT-U	ACAAATTAGGAACATTTTGGCCT
A13G1883-RT-L	GCCTTGTTGCTTTGCCTAAA
D03G0021-RT-U	ATGGAGCTATTTTGGATGCAGT
D03G0021-RT-L	AACTGGAGCACCAGAATTGG
D05G1179-RT-U	AGAGTCCTTTGGGCTCAAGT
D05G1179-RT-L	TTTGCTACAACTGACACCCC
D05G2362-RT-U	GAGGATCGAAGGGGAAGAGT
D05G2362-RT-L	AACTCACTCTGCGTTGTCTC
D06G0402-RT-U	TGTAGGACAGTTTCACGCTG
D06G0402-RT-L	GTCGACATAGGGACTGATGC
D10G1386-RT-U	GTTGAAGAGGTGGTGACAGG
D10G1386-RT-L	CTGGAACTACGTTCTCTGGC
